# Antitumor and antioxidant effects of *Clinacanthus nutans* Lindau in 4 T1 tumor-bearing mice

**DOI:** 10.1186/s12906-019-2757-4

**Published:** 2019-11-29

**Authors:** Nik Mohd Afizan Nik Abd Rahman, M. Y. Nurliyana, M. N. F. Natasha Nur Afiqah, Mohd Azuraidi Osman, Muhajir Hamid, Mohd Azmi Mohd Lila

**Affiliations:** 1 Department of Cell and Molecular Biology, Faculty of Biotechnology and Biomolecular Sciences, Universiti Putra Malaysia, 43400 Serdang, Selangor Malaysia; 2 Institute of Tropical Forestry and Forest Products, Universiti Putra Malaysia, 43400 Serdang, Selangor Malaysia; 3 Department of Microbiology, Faculty of Biotechnology and Biomolecular Sciences, Universiti Putra Malaysia, 43400 Serdang, Selangor Malaysia; 4 Department of Veterinary Pathology and Microbiology, Faculty of Veterinary Medicine, Universiti Putra Malaysia, 43400 Serdang, Selangor Malaysia

**Keywords:** Antitumor, Antioxidant, *Clinacanthus nutans* Lindau, Breast tumor

## Abstract

**Background:**

*Clinacanthus nutans* Lindau (*C. nutans*) is a species of in Acanthaceae family and primarily used in South East Asian countries. *C. nutans* is well known as Sabah snake grass in Malaysia, and its leaves have diverse medicinal potential in conventional applications, including cancer treatments. On the basis of literature search, there is less conclusive evidence of the involvement of phytochemical constituents in breast cancer, in particular, animal tumor models. The current study aimed to determine the antitumor and antioxidant activities of *C. nutans* extract in 4 T1 tumor-bearing mice.

**Methods:**

*C. nutans* leaves were subjected to methanol extraction and divided into two different concentrations, 200 mg/kg (low-dose) and 1000 mg/kg (high-dose). The antitumor effects of *C. nutans* extracts were assessed using bone marrow smearing, clonogenic, and splenocyte immunotype analyses. In addition, hematoxylin and eosin, tumor weight and tumor volume profiles also used to indicate apoptosis appearance. Serum cytokine levels were examined using ELISA assay. In addition, nitric oxide assay reflecting antioxidant activity was performed.

**Results:**

From the results obtained, the methanol extract of *C. nutans* leaves at 200 mg/kg (*P* < 0.05) and 1000 mg/kg (*P* < 0.05) showed a significant decrease in nitric oxide (NO) and malondialdehyde (MDA) levels in the blood. On the other hand, *C. nutans* extract (1000 mg/kg) also showed a significant decrease in the number of mitotic cells, tumor weight, and tumor volume. No inflammatory and adverse reactions related to splenocytes activities were found in all treated groups of mice. Despite its promising results, the concentration of both *C. nutans* extracts have also reduced the number of colonies formed in the liver and lungs.

**Conclusion:**

In conclusion, *C. nutans* extracts exert antitumor and antioxidant activities against 4 T1 mouse breast model with no adverse effect and inflammatory response at high dose of 1000 mg/kg, indicating an effective and complementary approach for cancer prevention and treatment.

## Background

Cancer is the uncontrolled development of abnormal cells that distinguish between benign and malignant tumors in which colon and breast cancer cases are among prevalent cancers diagnosed in humans. In Malaysia, the Ministry of Health (MOH) has reported that the mortality rate due to cancer cases has increased by approximately 10–11% throughout in 2016 [1]. The increased in the number of cancer cases has led to early death worldwide. The most common types of cancer treatment are surgery, hormonal therapy, chemotherapy, and radiation therapy. Beside modern treatments, herbal medicine uses plants or mixtures of plant extracts to treat diseases and promote health, since they contain various medicinal phytochemicals [2]. The natural sources have been recognized for tropical centuries and as an alternative and complementary approach to cancer treatments with minimal cost and side effects [3]. Medicinal plants with anticancer and antioxidant properties are *Annona muricata Linn* [4], *Andrographis paniculata* [5], *Gynura sarmentosa* [6], *Centella asiatica* [7], and *Clinacanthus nutans* [8, 9].

*Clinacanthus nutans* Lindau (*C. nutans*) is a species of the Acanthaceae family and traditionally known as Sabah Snake Grass in Malaysia. The plant has numerous therapeutic potentials in modern and traditional herbal medicine such as anti-diabetic [10], anti-inflammatory [11], anti-microbial [12], anti-viral [13], skin rashes and gout [14]. Many different parts of *C. nutans* are useful for cancer treatments such as aerials, seeds, flower, leaves and stems. Nevertheless, most of the previous studies used the plant leaves using various extraction protocols such as alcoholic, chloroform, petroleum and methanolic. The leaves are flat, opposite and narrowly elliptical-oblong, containing terpenoids and phenolic compounds [15]. *C. nutans* extracts have been used for the treatments of various types of carcinomas including colon cells, breast cells and brain cells [14, 16]. Ng et al. have also reported that *C. nutans* water extract induces human oral squamous cell apoptosis [17]. Despite claims regarding its antioxidant and anticancer capacity, several specifics yet to be examined, especially in animal models. Therefore, the present study was intended to determine the antitumor and antioxidant potentials of methanol extract of *C. nutans* in breast cancer cell line in vivo.

## Methods

### Chemicals and reagents

Murine mammary carcinoma cell line, 4 T1 cells and RPMI-1640 were purchased from American Type Culture Collection (ATCC). Griess Reagent Kit was purchased from Molecular Probes, Eugene, OR. DuoSet ELISA Development System was purchased from R&D Systems, USA.

### Plant materials

*Clinacanthus nutans* Lindau (*C. nutans*) was collected from TKC Herbal Nursery Sdn Bhd, Negeri Sembilan, Malaysia, and was classified as the whole plant of *C. nutans* by a science officer named Mr. Lim Chung Lu from the Phytomedicinal Herbarium at Institute of Bioscience (IBS), UPM before the sample was deposited in our laboratory (Voucher No. SK2775/15).

### Preparation of extract

The *C. nutans* leaves were harvested freshly. After the leaves have been thoroughly dried, the leaves were then ground into powder form and sequentially soaked in methanol at room temperature. The extract was then filtered using Whatman42 filter paper. The filtrate was oven-dried at 37 °C and then kept at 20 °C until further analysis.

### Cell culture

Murine mammary carcinoma cell line, 4 T1 cells were purchased from the American Type Culture Collection (ATCC, USA) and were maintained in RPMI-1640 medium supplemented with 1% penicillin-streptomycin, 1 mM sodium pyruvate, 2 mM glutamine and 10% FBS. Cells were placed in a CO_2_ incubator with 95% humidity at 37 °C.

### Animal

Six to eight-week-old female BALB/c mice were purchased from the Animal House of Faculty of Veterinary Medicine, Universiti Putra Malaysia. The mice were kept under a condition of 12-h dark/light cycle at 25 °C. The mice were fed with standard pellet diet and distilled water ad libitum. All the procedures involving mice were carried out in compliance with the regulations of the Animal Care and Use Committee (ACUC; UPM/IACUC/AUP-R086/2017), Universiti Putra Malaysia.

### Tumor inoculation and treatments

Mice were divided into 4 groups (*n* = 7), which consisted 1) control (without breast cancer, untreated), 2) untreated (with breast cancer, untreated) and two other groups of mice harboring breast cancer treated with 200 mg/kg (low-dose) and 1000 mg/kg (high-dose) of *C. nutans* extract. Similar doses were reported with normal behavior in a study by Lau et al., [18]. In this animal model, 1 × 10^6^ 4 T1 cells were subcutaneously inoculated, and 10 days of incubation were given for tumor growth prior to initiation of treatment. All treatments were administrated daily by oral gavage for 28 days. The size of tumors was measured using a vernier caliper with following the formula, V = (W x W x L)/2 where V is volume, W is width and L is length. After 28 days, mice were euthanized by cervical dislocation. Blood, tumors and vital organs such as liver, lung, spleen, bone marrow were collected for the following analyses. The weight of the tumors was recorded as well.

### Immunotyping of Splenocytes

After mice euthanasia, the spleen was aseptically collected and excised for isolation of splenocytes for immunophenotyping as defined in the previous study [19]. Briefly, the spleen was prepared into single-cell suspension with Hank’s Balance Salt Solution (HBSS) containing 5 mM HEPES and 10% FBS by meshing with a 70 μm strainer. The splenocytes were incubated in lysis buffer (0.1 mM EDTA, 10 mM KHCO3, 0.15 M NH4Cl at pH 7.5) for 10 min at 4 °C for the removal of red blood cells. The cells were then rinsed with PBS before staining with appropriate antibodies (Abcam, USA) at 37 °C for 3 h. Next, the cells were rinsed with PBS twice before fixed with 1% paraformaldehyde (PFA). The cells were kept in the dark at 4 °C until analyzing through FACSCalibur flow cytometer (BD, USA).

### Clonogenic assay of lung and liver

The procedure has already been identified and carried out with minor modifications [20]. Briefly, the lung and liver were harvested and then cut into small fragments in sterile condition. The fragments were incubated in 5 ml enzyme cocktail containing 1X PBS, 1 mg/ml of hyaluronidase and 1 mg/ml of type I collagenase for 30 min at 37 °C. After incubation, the cell-containing solution was passed through a 70 mm cell strainer. The cells were pelleted down and washed with PBS twice. The cells were resuspended in 10 ml selection medium and incubated for 7 to 10 days at 37 °C, 5% CO_2,_ and 95% humidity. After incubation, the plate was prepared with fixing of methanol and stained with crystal violet to count the number of colonies formed per organ.

### Bone marrow smearing

Bone marrow smears were prepared from the contents of the right femur, as previously described [21]. Briefly, bone marrows were flushed with 1X PBS and smeared across a clean glass slide. The slide was dried in air at room temperature prior to fixation with 100% methanol for 30 min and air-dried again before staining. The slide was then stained with Giemsa stain for 10 min and then air-dry.

### Hematoxylin and eosin staining (H&E)

Formalin-fixed paraffin-embedded sections of the tumor tissues were carried out as described in our previous study [22]. Briefly, the tumors were harvested and fixed in 10% neutral buffered formalin before being sent to the Histopathology Laboratory, Faculty of Veterinary Medicine, Universiti Putra Malaysia for hematoxylin and eosin staining. The stained tissue sections of 0.45 μm were examined under a microscope (Nikon).

### Nitric oxide (NO) radical scavenging assay

The level of nitric oxide production was detected using the modified Griess assay [23]. Briefly, a mixture of 20 μl Griess reagent, 150 μl nitrite-containing sample and 130 μl distilled water were prepared. On the other side, a photometric reference sample was prepared by mixing 20 μl of Griess reagent and 280 μl of distilled water. The nitrite solutions were prepared by diluting the standard solution with distilled water. The absorbance of generic nitrite solutions was then measured in order to plot a standard curve of nitrite concentration against absorbance. The concentrations of nitrite corresponding nitrite concentrations corresponding to the absorbance of the samples were read from the standard plot.

### Malondialdehyde (MDA) assay

For quantification of MDA level, this procedure was adapted from the procedure by Samiaa et al., [24]. A mixture of 200 μl sample, 800 μl of PBS, 25 μl of butylated hydroxytoluene (BTH) and 500 μl of trichloroacetic acid (TCA) was prepared in a 50 ml tube and incubated on ice for 2 h. After incubation, the tube was centrifuged for 15 min at room temperature prior to mixing 1 ml of the supernatant with 75 μl of 0.1 M EDTA and 250 μl of thiobarbituric acid (TBA) in 1 M NaOH. The mixture was boiled for 15 min and cooled down to room temperature. The absorbance was measured at 532 nm and 600 nm using a spectrophotometer (Beckman Coulter, USA).

### Cytokines ELISA assay

The level of IL-2 and Interferon-γ (IFN-γ) secretions were assessed from the serum mice. The samples were collected and analysed using the DuoSet ELISA Development System (R&D Systems, USA). Designated capture antibodies (Mouse IL-2 Capture Antibody and IFN-γ Capture Antibody) were diluted to working concentration in PBS without carrier protein. The procedures were performed in accordance with the manufactural protocol. Briefly, 96-well plates were coated with 100 μl per well of the diluted Capture Antibodies and incubated overnight at room temperature. The following day, the plates were washed three times with Wash Buffer. The plates were then blocked with Block buffer at room temperature for 1 h. Next, 100 μl of serum in Reagent Diluent was added in each well and incubated for 2 h. The solutions were aspirated, washed three times and added 100 μl of detection antibodies were applied. The plates were then incubated for another 2 h. Next, each well was incubated with Streptavidin-HRP at room temperature for 20 min. The plates were washed three times before incubation with Substrate Solution for 20 min. Lastly, the reaction was stopped by adding Stop Solution and the plates were read at 450 nm and 570 nm using a microplate reader (Azure Biosystems).

### Statistical analysis

All data presented in the standard error of the mean (SEM) and performed using the SPSS version17. Data were analyzed using one-way ANOVA, followed by Dunnett’s multiple comparisons test. *P* < 0.05 was considered to be significant.

## Results

### Tumor growth and tumor weight

**Fig. 1:**
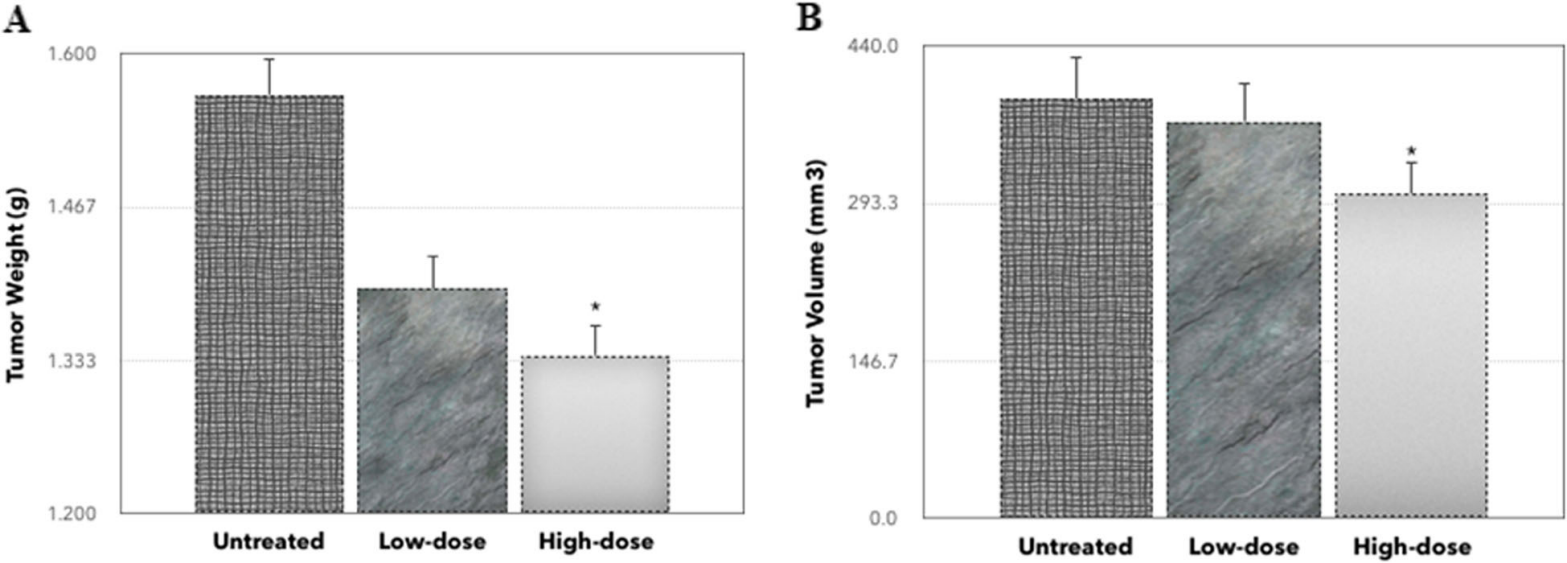
The weight and volume of tumors from untreated and *C. nutans*–treated groups. **a** Weight of tumors was measured after being harvested on 28 days of post-treatment. **b** Volume of tumors was measured using a Vernier caliper. Each value represents the mean ± standard error of the mean. Significance is set at *P < 0.05

After 28 days of treatment, the high-dose of methanol extract of *C. nutans* significantly reduced the volume and weight of the tumor in mice. The weight of tumors was reduced in the low-dose group (1.396 ± 0.251 g) and high-dose group (1.338 ± 0.327 g) compared to the untreated group (1.565 ± 0.357 g) as rendered in Fig. 1a. Similarly, in Fig. 1b, the volume of tumors in the untreated group was 391.0 ± 26.7 mm^3^, whereas in the low-dose of treatment group, the weight decreased to 370.1 ± 24.9 mm^3^. Tumor volume also significantly decreased from 391.0 ± 26.7 mm^3^ to 302.2 ± 40.3 mm^3^ in the high-dose of *C. nutans*–treated group.

### *C. nutans* affects immune cell populations

**Fig. 2:**
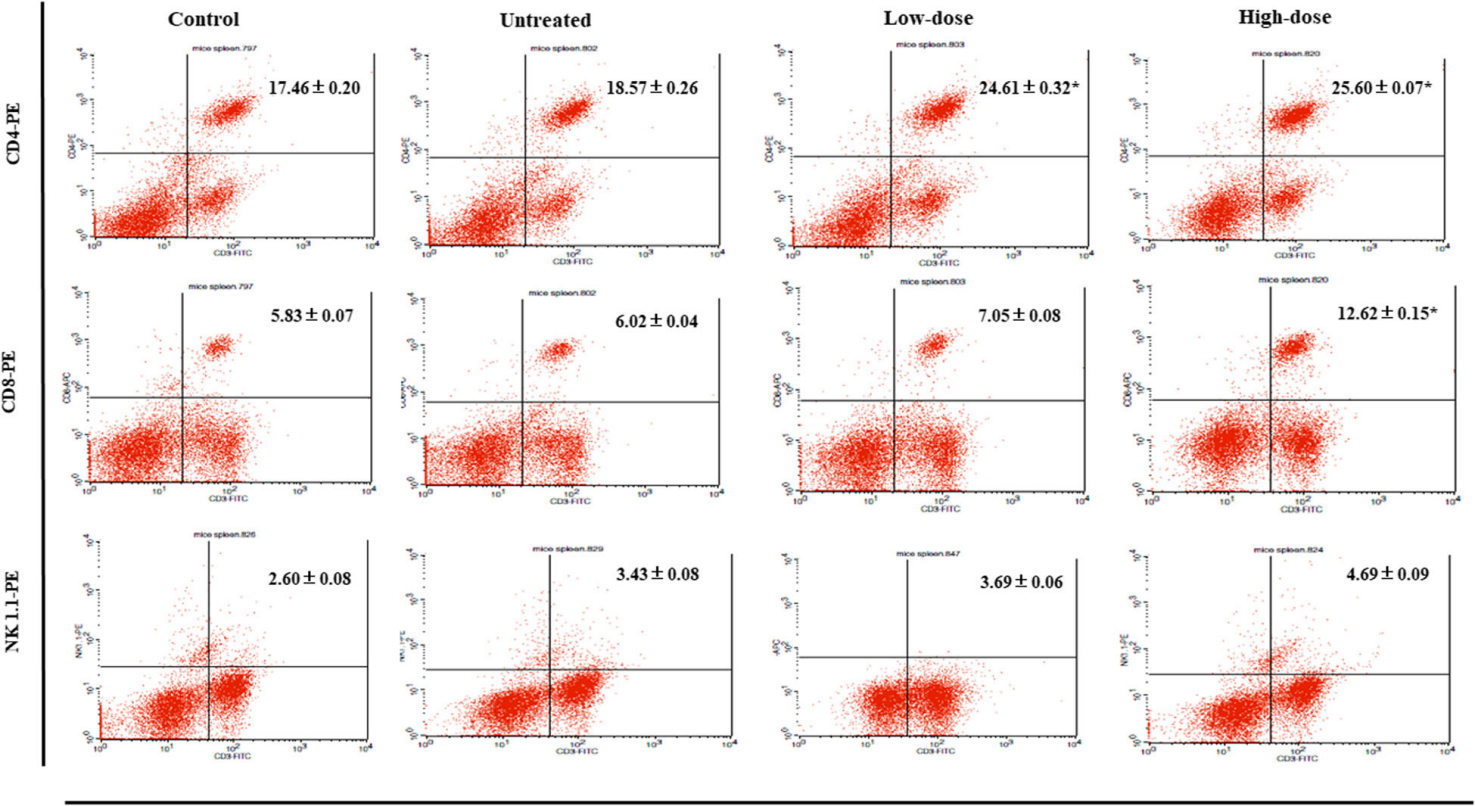
Flow cytometry analysis of immune markers (CD4, CD8, CD3 and NK 1.1) on the splenocytes of the untreated mice, treated mice with low- and high-dose of methanol *C. nutans* extract. The percentage of the CD4/CD3 T-cell and CD8/CD3 T-cell population was increased significantly for both low and high-dose of treatment when compared to the untreated and control groups. The population of natural killer (NK) 1.1/CD3 cells was also increased in both low and high-dose of *C. nutans* treatment when compared to untreated and control groups

**Fig. 3:**
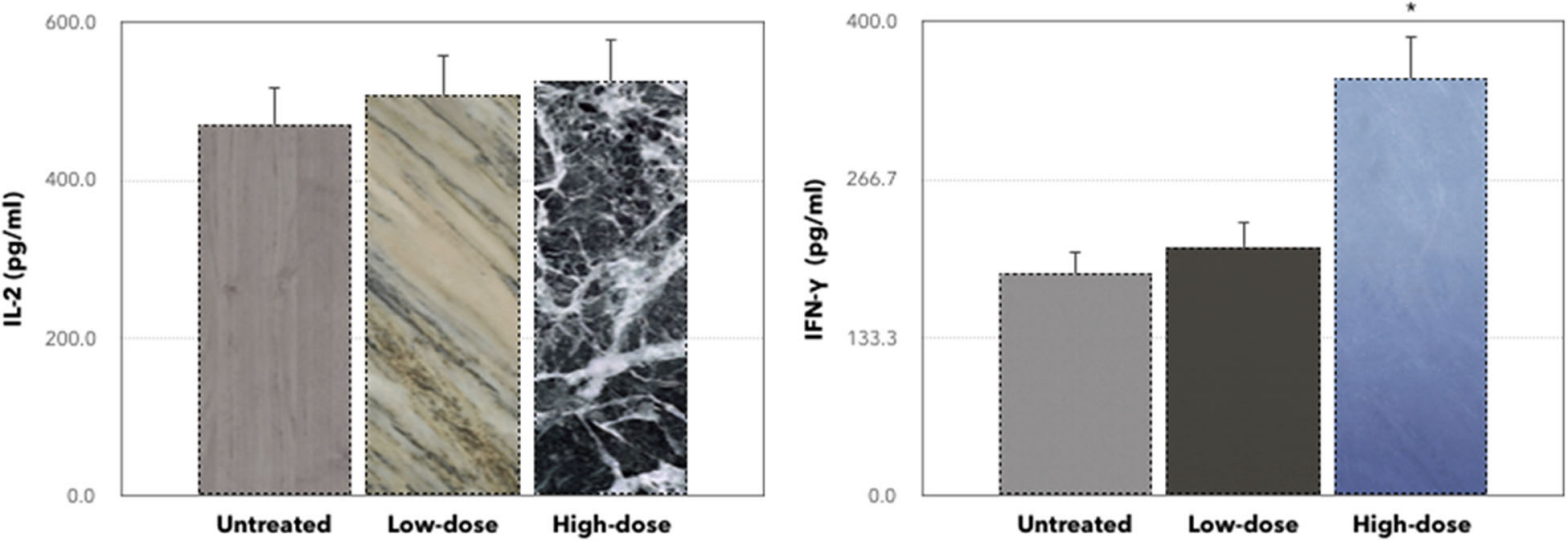
Enzyme-linked immunosorbent assay analysis on the detection of the level of in IL-2 and IFN-γ in serum of the untreated group, treated mice with low and high-dose of *C. nutans* extract. The levels of expression for both IL-2 and IFN-γ have increased for both low and high *C. nutans* treatment when compared with the untreated group. Each value represents the mean ± standard error of the mean. Significance is set at *P < 0.05

Several antibodies for immune system marker antibodies (CD3, CD4, CD8, and NK1.1) were used to identify the *C. nutans* activity on splenocyte cell populations. As rendered in Fig. 2, the population of CD4/CD3 and CD8/CD3 cells was found to increase significantly, independent of low-dose and high-dose of *C. nutans* treatments compared to the untreated group. There was also a similar pattern in the NK1.1/CD3 population in low-dose and high-dose treatments relative to the untreated group even not significantly different. To further refine the anti-metastatic effect of *C. nutans*, the secretion of cytokines (IL-2 and IFN-γ) were measured, as conferred with Fig. 3. The expression levels of IL-2 and IFN-γ have increased in both low-dose and high-dose of *C. nutans* treatments compared to the untreated group. As rendered in Fig. 3, the expression level of IL-2 was fewer in the untreated group (471.1 ± 46.3 pg/ml) compared to low-dose and high-dose of *C. nutans* groups were 508.9 ± 34.2 pg/ml and 525.7 ± 28.5 pg/ml, respectively. In addition, the level of IFN-γ in the untreated group (187.2 ± 3.24 pg/ml) was decreased to 209.7 ± 12.3 pg/ml and 352.4 ± 24.9 pg/ml in the low-dose- and high-dose-treated mice.

### *C. nutans* regulates inflammation and antioxidant activity

**Fig. 4:**
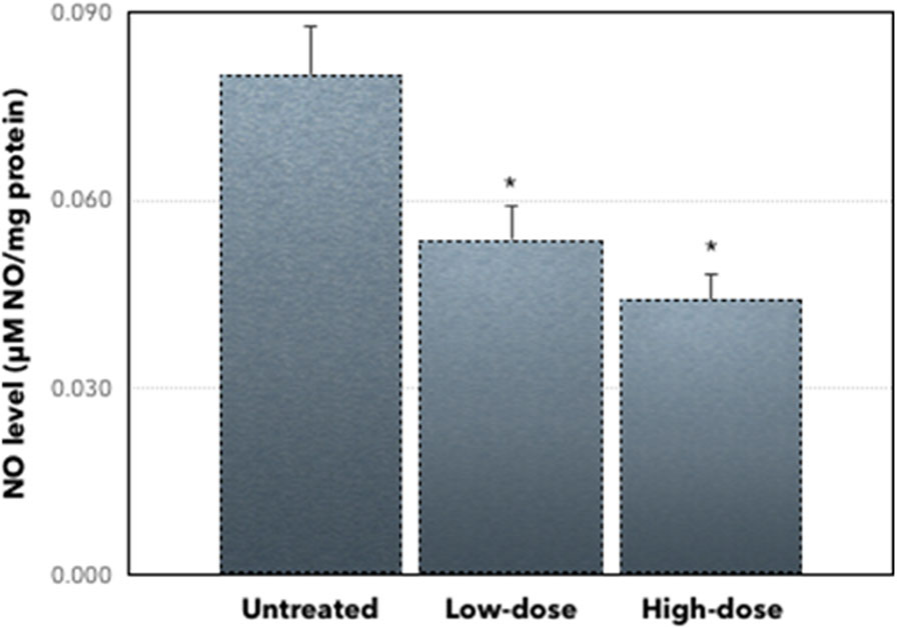
Level of nitric oxide assay from the untreated and treated groups (low-dose and high-dose of *C. nutans*). Each value represents the mean ± standard error of the mean. Significance is set at *P < 0.05. The level of NO decreased significantly in low-dose and high-dose of treatment compared to the untreated group

**Fig. 5:**
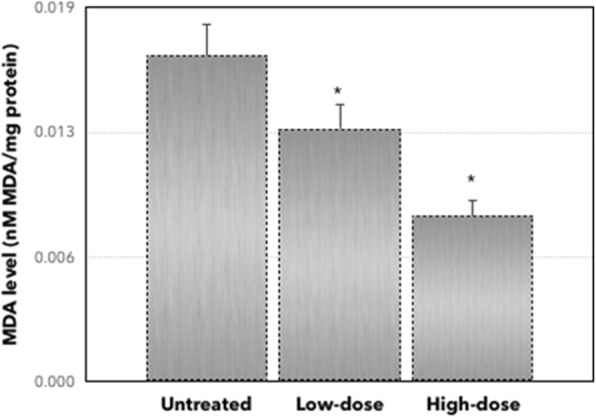
Level of MDA from the untreated and treated groups (low-dose and high-dose of *C. nutans*). Each value represents the mean ± standard error of the mean. Significance is set at *P < 0.05. The level of MDA decreased significantly in low-dose and high-dose of treatment compared to the untreated group

To elucidate the inflammation effect of *C. nutans* in the tumors, the level of nitrite oxide (NO) was measured. The level of NO was decreased in both low-dose and high-dose of *C. nutans* treatment groups compared to the untreated group. As presented in Fig. 4, the expression level of NO was decreased from 0.080 ± 0.025 μM/mg in the untreated group to 0.054 ± 0.013 μM/mg in low-dose of treatment and 0.044 ± 0.010 μM/mg in high-dose of *C. nutans* treatment. To determine the antioxidant properties of *C. nutans* methanol extract, while, the level of MDA was also elucidated. As shown in Fig. 5, the MDA level in the untreated group was 0.017 ± 0.001 nM/mg, whereas in the low-dose and high-dose of *C. nutans* treatments were 0.013 ± 0.001 nM/mg and 0.0083 ± 0.001 nM/mg, respectively.

### *C. nutans* possesses an anti-metastatic effect in vivo

**Fig. 6:**
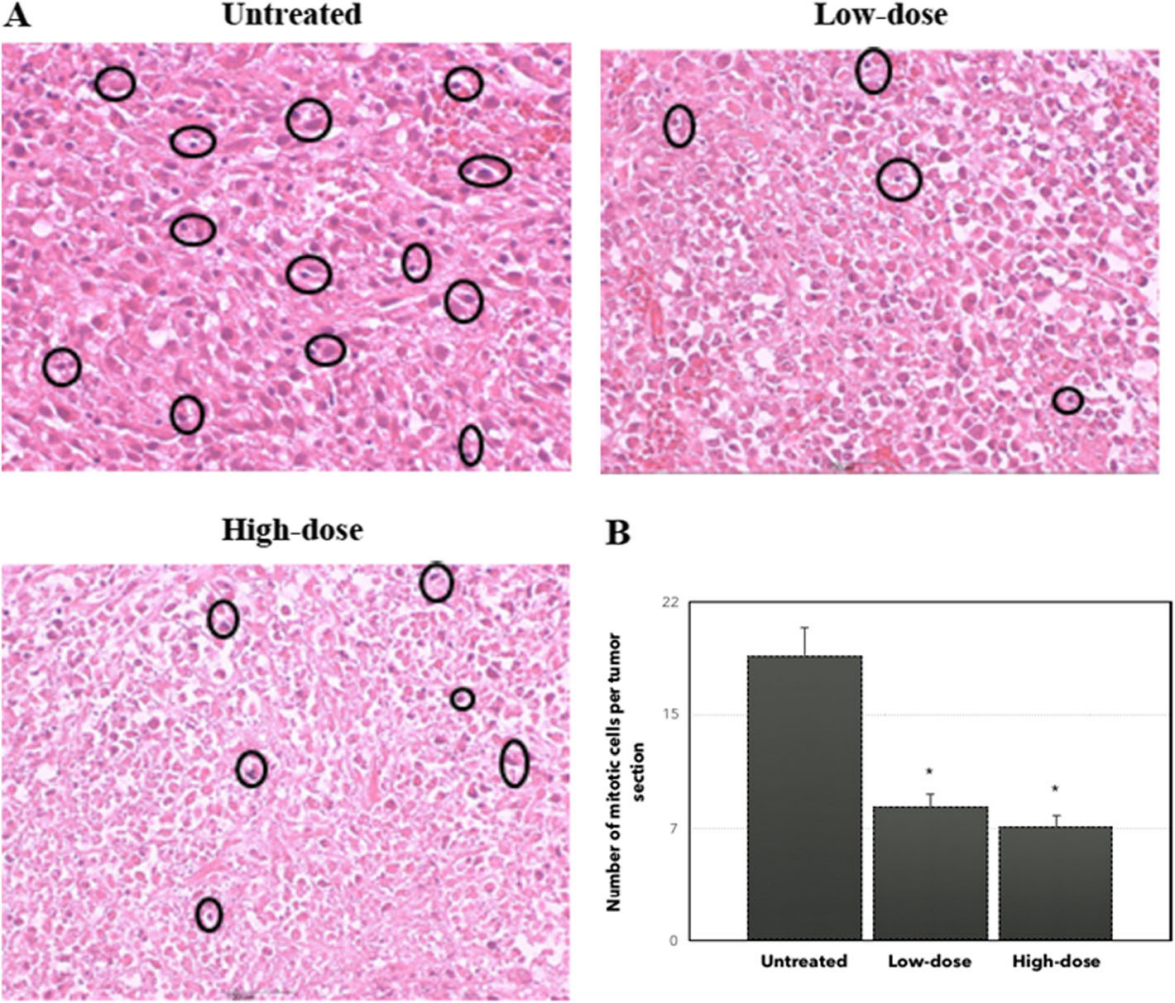
Histology analysis of the untreated, low-dose and high-dose of *C. nutans*. **a** Both tumor samples of the untreated and treated groups are stained with hematoxylin and eosin (H&E). (**b**) The number of mitotic cells decreased significantly in low-dose and high-dose of *C. nutans* treatment compared to the untreated group. Notes: **a** Circles represent cells undergoing mitosis. Magnification: 40X. Significance is set at *P < 0.05

**Fig. 7:**
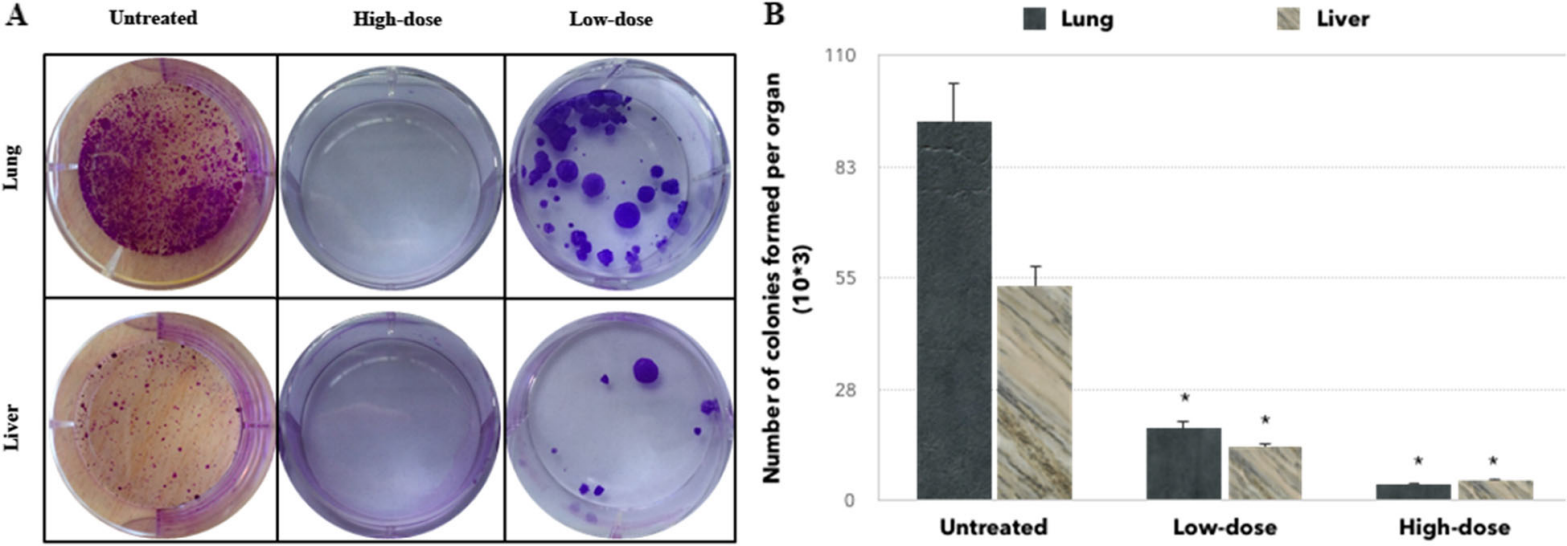
Clonogenic assay of mice organs. **a** Representative images of colonies formed in the lung and liver organs. **b** Bar chart of the total 4 T1 colonies formed from the mashed lung and liver harvested from the untreated, treated mice with low-dose and high-dose of *C. nutans* treatment after 10 days of incubation. Notes: **a** Lung, dilution factor: 10^3^; liver, dilution factor: 10^3^. **b** Each value represents mean ± standard error of the mean; *P < 0.05

**Fig. 8:**
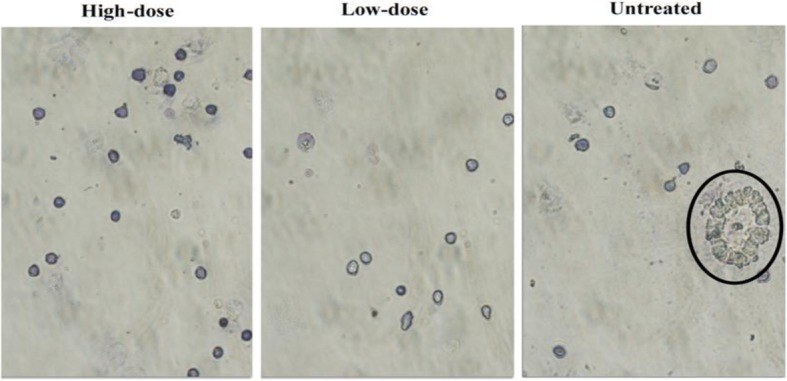
Bone marrow cells stained with Giemsa viewed under a phase-contrast microscope. Notes: Circles indicate the presence of abnormal cells base on the different morphology. Magnification: 40X

To determine the anti-metastatic activity of the *C. nutans* in vivo, the tumor sections were stained with hematoxylin and eosin (H&E). As rendered in Fig. 6, abnormal mitotic figures and coarse chromatin data were seen more visible and frequently in the untreated group compared to the low-dose and high-dose of treatment. In addition, the number of mitotic cells decreased in both the low-dose and high-dose of treatment as shown in Fig. 6b. On the other hand, the clonogenic assay was established to elucidate the anti-metastatic properties of *C. nutans* further. As shown in Fig. 7, the number of colonies produced in the liver and lung with a low-dose of treatment was significantly decreased while no colonies were formed at a high-dose of treatment. The presence of large and irregular cells was reported as metastatic cells in the bone marrow assay as shown in Fig. 8 which has been found only in the untreated group.

## Discussion

Previous studies have reported that the *C. nutans* extracts possess anticancer and antioxidant properties in various cancer cell lines in vitro; nevertheless, their antioxidant and antitumor activities have not been completely elucidated in murine models [25–27]. Therefore, our experiments have shown that the methanolic extract of *C. nutans* leaves could inhibit the tumor progression in 4 T1 tumor-bearing mice model. Mean weight and tumor size were significantly reduced after 28-days of *C. nutans* treatments. These findings were showed as typical phenotypic features of apoptosis, and similar patterns were also reported the inhibitory benefits using water and petroleum ether protocols [28, 29]. In response to DNA damage, a typical appearance of actively mitotic cells in *C. nutans*-treated tumors was decreased compared to control and untreated groups (H&E profiles). Hence, targeted inhibition of tumors would generally affect only active mitotic cells [30]. Taken together, these results suggested that the progression of 4 T1 tumors hindered by apoptosis response.

Immune-mediated responses in malignancy are unique and diverse, such that the interfering of immune cell populations at different stages of tumor progression might be affected by the tumor aggressiveness [31]. Since T-cells are a pivotal player in the tumor microenvironment, promoting their function might have adverse effects on solid tumors. It was evident that the *C. nutans* extract increased several types of NK1.1 cell and T-cell populations. The percentages of CD3, CD8, and NK1.1 cells have increased in the *C. nutans-*treated groups (low and high-doses) compared to the untreated group. Both T-cells and NK cells activity contribute to the eliminating of tumor cells by inducing cell lyses. The cytotoxic T-cells (CD4 and CD8 cells) and NK cells played a critical role in the surveillance and characterized their elimination of target cells [32, 33]. Thus, increasing of the activities of immune cells such as CD3, CD4, CD8, and NK1.1 thereby confer increased the phytochemical constituents efficacy, resulting in impaired tumor metastasis and progression. Furthermore, increased CD3 and CD8 cells are also associated with improved cancer survival rates.

Several studies suggest that cytokines also play significant roles in the regulating of immune cells [34, 35]. For instance, IL-2 is necessary for T-cell activation and contributes to its clinical benefits, such as in controlling the survival of immature and mature T cells [36]. Therefore, the increased IL-2 secretion may contribute to the capacity of T cells activity, thus boosting the immune system. Furthermore, the level of IFN-γ secretion also increased in the *C. nutans*-treated mice. As a consequence, the activation of NK cells and CD8 cells with stimulation of other cytokines (IL-2 and IFN-γ) have contributed to synergy impact on the inhibition of tumor cells. Cytokines involved in cancer-related inflammation represent a potential target and innovative diagnosis for clinicians and scientists. A previous study showed that IL-6 has the ability to induce apoptosis in many human ovarian cancer developments by blocking the IL-6R/STAT3 pathway [37].

The correlation between inflammation and cancer in the tumor microenvironment has been extensively studied. Indeed, inflammation can promote oncogene activation leading to tumor initiation, tumor progression and metastatic dissemination in the body [38, 39]. Nitric oxide (NO) is one of the short-lived signaling molecules which act as an intercellular messenger in various immune and inflammatory conditions [40]. *C. nutans* treatments result in the decline of NO levels in both the low-dose and high-dose groups. The results of the NO were consistent with malondialdehyde (MDA) levels in the tumor tissues. The MDA levels within the tumors are reduced when treated with *C. nutans* due to its antioxidant properties. Similarly, the antioxidant effect in hepatoma cells was also exhibited by decreasing the MDA levels. According to the findings, it could be advocated that *C. nutans* may protect cancer cells from apoptosis signals and facilitate the survival of tumor cells [41, 42]. It also protects healthy cells and their cellular mechanism from the damage caused by unstable molecules known as free radicals [43].

Tumor angiogenesis is a new development of blood capillaries/vessels which tumor cells can migrate in the blood or lymphatic system and circulate through the intravascular during metastatic progression. The versatile platform may provide a chance of cancer cells to spread and develop in new areas distant from their primary tumors [44]. The inhibition of tumor angiogenesis and inflammation-related markers by *C. nutans* have reflected the reduction of the number of colonies established in the liver and lung. Therefore, the finding showed that the *C. nutans* extracts have effectively inhibited the metastatic potential of the 4 T1 tumor-bearing mice. In addition, the findings of bone marrow smearing have also shown that no appearance of atypical or erratic cells were found in the *C. nutans*-treated mice.

## Conclusion

In conclusion, the methanol extract of *C. nutans* leaves even in low-dose (200 mg/kg) contains antitumor and antioxidant constituents that are capable of scavenging free radicals and inhibiting the growth of tumor progression. These findings also indicate that the phytochemical constituents present in methanol extract could be used as an alternative and complimentary for cancer prevention and treatment. However, more extensive studies are needed to characterize the bioactive constituents of the *C. nutans* extract and to understand the underlying mechanism of antitumor activity in order to unveil its potential use in cancer therapy.

## Data Availability

The datasets analyzed during the current study are available from the corresponding author on reasonable request.

## Abbreviations

FBS: Fetal bovine serum
H&E: Hematoxylin and eosin
IFN-γ: Interferon gamma
IL-2: Interleukin-2
IL-6R: Interleukin-6R
MDA: Malondialdehyde
STAT3: Signal transducer and activator of transcription 3
